# Furmidge Equation Revisited

**DOI:** 10.1021/acs.langmuir.5c01302

**Published:** 2025-05-02

**Authors:** Yotam Stern, Rafael Tadmor, Assaf Miron, Appu Vinod

**Affiliations:** Department of Mechanical Engineering, Ben Gurion University of the Negev, Beer Sheva 8410501, Israel

## Abstract

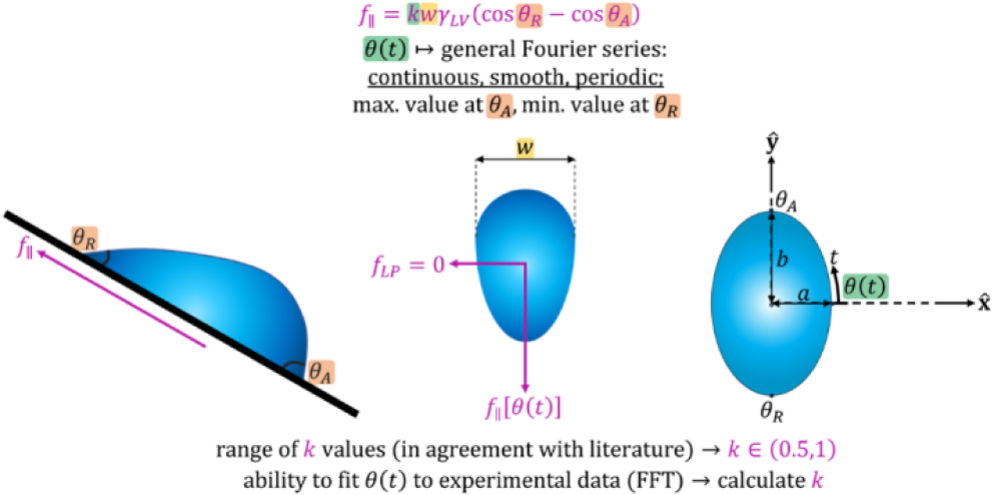

This paper addresses the issue of the discontinuous function that gives rise to the Furmidge equation, a long-standing problem in interfacial science. The force at the contact line of a sliding drop is related to the drop size, the contact angle hysteresis, the surface tension, and a geometrical prefactor *k* which depends on the distribution of the contact angle about the contact line. The most common expression for the contact line force, called the Furmidge equation, takes *k* = 1 and is based on a discontinuous contact line, while corrections to this model pose polynomial functional forms for the contact angle, engendering discontinuities in the derivatives. Moreover, experimental findings provide a wide range of *k* values for different drops on different solids, and this range is yet to be explained in a physical context. Owing to this, the understanding of forces on sliding drops remains lacking. We construct a general model based on a Fourier series, and we further generalize this model by superposing a series of Gaussian curves on our Fourier series. The result of this model is a range of *k* values, in accordance to the range of experimental values which appear in the literature. Additionally, we fit our functional form for the contact angle to experimental data and find good agreement, as well as good agreement between *k* values.

## Introduction

The motion of liquid droplets is at the center of many physical processes.^1−12^ A case of particular significance is the lateral parallel force acting on a sliding drop moving at a steady speed relative to the solid substrate on which it was placed, and an equation which has often been used to describe such forces is the often-called Furmidge equation.^13−28^ This equation was earlier found by Frenkel^29^ and Kawasaki,^14^ and in both cases of Furmidge and Kawasaki, the equation describes the force required to slow a sliding drop.^13,14^ As such this equation, which essentially describes the dynamic friction of drops, has often been used outside its original purpose to describe the onset of motion of drops. In addition, many studies “correct” this equation with some prefactor *k* which they determine in order to reconcile their experimental/numerical results.^15−28^ The results vary widely between 0.5 and near or above 1, showing an apparently irreconcilable difference between the minimal and maximal values of *k*. This presents a significant challenge in applying these results in a scientific or engineering context.

In this paper, we obtain the Furmidge-Kawasaki equation for a drop sliding at a steady speed through a more physically consistent approach, which allows explaining the discrepancies obtained experimentally. Unlike previous theoretical studies which each predict a single specific *k* value, we find a range of physical values which *k* can take for a spectrum of reasonable geometries, while comparing our range to experimental data from the literature and explaining how apparent values outside this range may be obtained experimentally. We also model the distribution of the contact angle along the triple line, connecting between various models and the results they yield.

Our approach, like those of many previous authors,^13,15,16,19^ does not involve solving the full (and coupled) hydrodynamic problem as posed by some previous studies.^17,30^ To tackle the hydrodynamic problem, these authors assume specific boundary conditions for the speed of the advancing and receding contact lines which depend on the advancing and receding contact angles, respectively, and a no slip condition at the solid–liquid interface, thereby defining the specific systems for which it would be valid. Similarly, our approach is different to the approach of quasi-2D droplet models, whose simplified geometry allows solving the fluid dynamic equations in a more tractable manner.^31,32^ Meanwhile, the advantage of the approach presented in this paper is that it assumes certain functional forms which are physically feasible and shows that these forms, at the very least, approximate the general case, regardless of the solid–liquid interaction. At the same time, in comparison to the Furmidge-Kawasaki model and its various modifications, we expand on the currently available predictive capabilities of assumed functional forms of the contact line. A further advantage of the expanded range of *k* values obtained here is that, on performing an experiment/simulation, one could see if their obtained value of *k* is physically feasible or not, facilitating the validation process.

## Theoretical Methods

### Geometry and Forces

**Figure 1:**
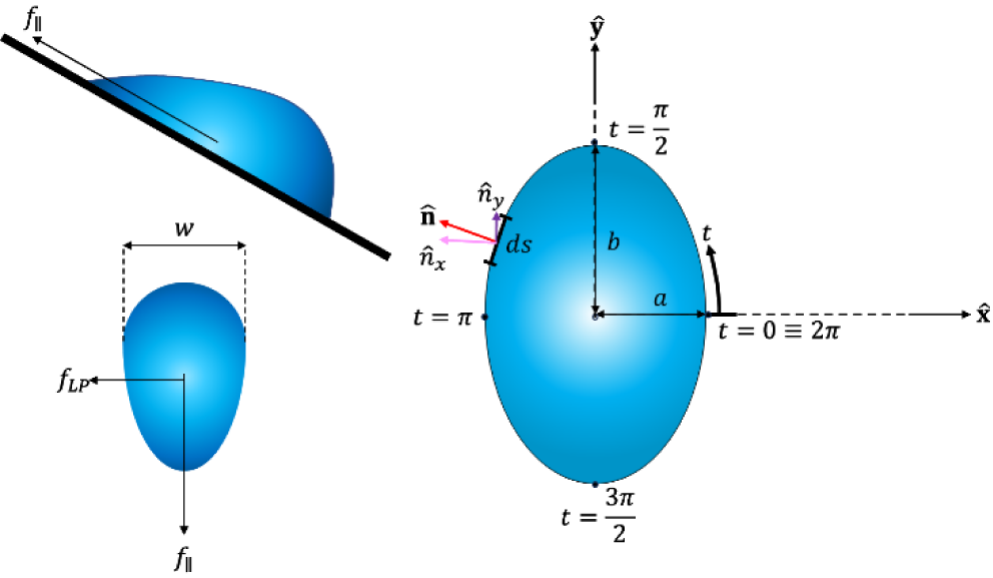
Top-left: schematic of the system where a liquid drop slides down a solid incline. Bottom-left: a force, *f*_∥_ acts against the direction of motion while another force, *f*_LP_, is labeled to act in the direction perpendicular to motion along the plane of the solid. Right: a top view of a drop with an elliptical triple line with semiminor and semimajor axes *a*,*b*, respectively. The **x̂,ŷ** components of the normal direction are shown, as is the sector length. In addition, we show the coordinate system, including the parameter *t* and its values at the front, back, and sides of the drop.

We consider drops placed on smooth solid surfaces which are then tilted incrementally until sliding begins. Once the drop begins to slide, the tilt of the solid surface is fixed so that the sliding is steady, infinitesimally above the critical inclination angle required for the drop to slide. We neglect the effect of cracks and chemical impurities or blemishes on the sliding and assume the drop to be nonvolatile. Since we are focusing on steadily sliding drops (not on pinning/depinning), the initial shape and dynamics corresponding to placing the drop on the substrate are not considered. Figure 1 shows the geometry of the system schematically, where the drop has a width of *w*. A lateral parallel force *f*_∥_ and lateral perpendicular force *f*_LP_ act on the drop, where *f*_LP_ is not the normal force but rather a force parallel to the solid and perpendicular to the direction of motion (and its sum along the drop’s circumference is expected to be 0).

As shown in Section S1 of the Supporting Information, a drop in equilibrium on a smooth, flat surface has a circular contact with the surface.^10,33−35^ Furthermore, there is an equilibrium contact angle such that
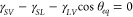
1where γ*_SV_*,γ*_SL_*,γ*_LV_* are the solid–vapor, solid–liquid, and liquid–vapor surface tensions and *θ*_*eq*_ represents the equilibrium contact angle for the drop at rest.

However, a drop on a smooth, inclined, surface has an oblong and elongated contact,^13,17,36,37^ with the contact angle, *θ*, varying along the perimeter between the receding contact angle, *θ*_*R*_, to the advancing contact angle, *θ*_*A*_. Approximating this shape to an ellipse,^38^ we write
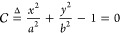
2where *a* = *w*/2,*b* = *l*/2 are half the width and half the length of the drop, respectively, as shown in Figure 1. We parametrize the ellipse by
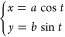
3

We can hence calculate the normal to the contour by^39^
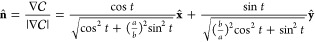
4which is also shown in Figure 1. Now, writing a force balance on a differential line element of the drop’s perimeter, we have
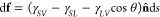
5awith . Resolving this into the parallel and perpendicular components, and plugging in the equilibrium contact angle from eq 1, we obtain
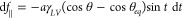
5b
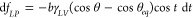
5c

Integrating eqs 5b and 5c, we get the lateral parallel and perpendicular forces *f*_∥_,*f*_*LP*_, given by
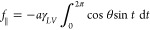
6a
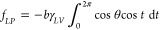
6bwhere we dropped the cos *θ*_*eq*_ terms here as .

According to experimental findings it may be more accurate to model the triple line shape by the union of two semiellipses.^15^ It is shown in Section S2 of the Supporting Information that modeling the geometry of the drop surface in such a way leads to the same results as found here, such that eq 5b remains unchanged in such a case. Furthermore, some previous studies have found that the contact geometry of sliding drops may include a cusp at the receding edge.^36,37,40,41^ We also deal with the repercussions of such cases in Section S3 of the Supporting Information and show that such cases in fact strengthen the rest of the results in this paper.

### Contact Angle Variations and Lateral Force Calculations

#### Previous Models: Furmidge-Kawasaki, Piecewise Polynomials

While the bulk hydrodynamics and the shape of the contact line are intimately tied, our approach resembles that of previous authors who assumed certain functional forms for the shape distribution of the contact angle along the contact line (referenced separately below). Meanwhile, in this section we analyze these forms and present certain limitations and/or nonphysical implications.

**Figure 2:**
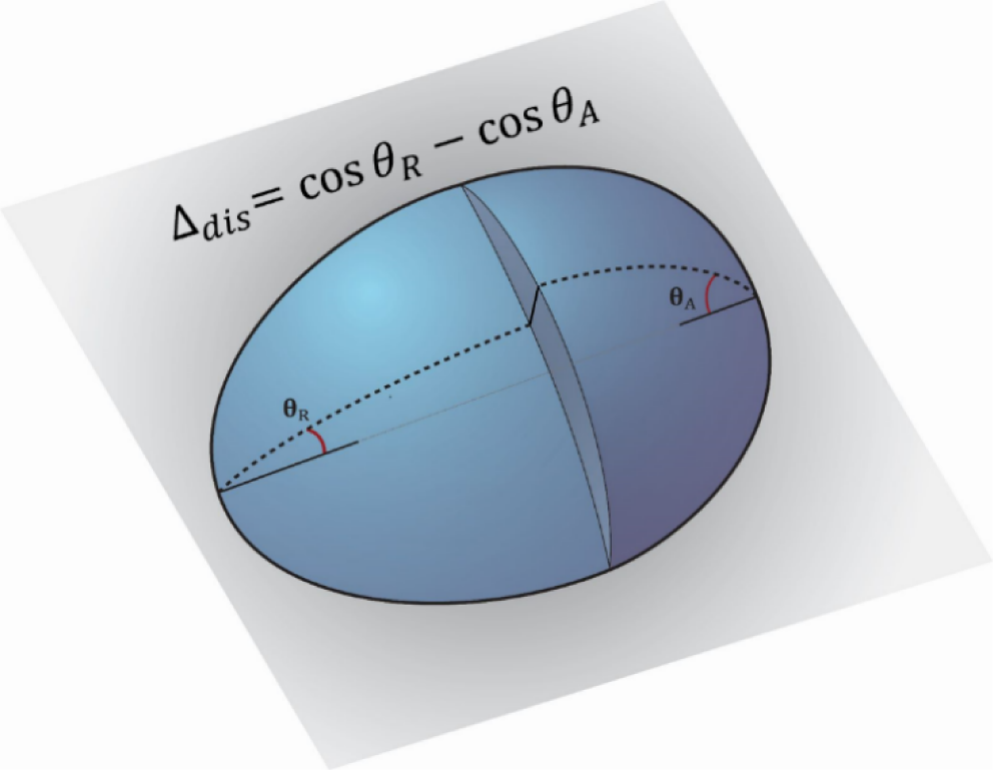
Contact angle model as assumed by Furmidge and Kawasaki. The model is inherently discontinuous at *t* = π, 0 ≡ 2π (see Figure 1) and is therefore unphysical as shown.

Furmidge and Kawasaki assumed a drop with two contact angles characterizing the advancing and receding sides,^13−15^ as demonstrated in Figure 2. This can be written as
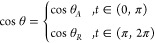
7

Combining eqs 5b and 7 and noting eq 7 is piecewise continuous, we get that
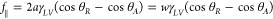
8a
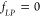
8bwhich shows how the Furmidge equation is obtained while the force balances in the perpendicular direction. The main issue with this model is the discontinuous nature of the drop model which is not physical. In fact, this model is twice discontinuous, once at *t* = π and again at *t* = 2π ≡ 0 where the drop should be periodic. Furthermore, while the local shear stress is already singular at the contact line,^15^ a discontinuity in the contact angle along the contact line implies a local energetic imbalance, contradicting the assumption of steady sliding.^10^ In such a case, the local shear stress would itself be a function of time until a smooth variation of the contact angle is obtained.

Due to the problematic nature of a discontinuous contact angle model, others have adopted a piecewise linear contact angle model.^16^ The boundary conditions of the advancing and receding contact angle, as well as that of symmetry with respect to the *y* axis, yield a piecewise linear function to describe the cosine of the contact angle by
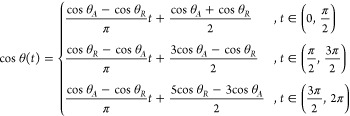
9where it is more convenient to define cos *θ* rather than *θ*. Combining with eq 5b, we get
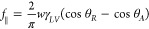
10a
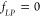
10b

This already shows that by making our model continuous we end up getting a prefactor which is less than 1. In this case, we obtained that *k* = 2/π. However, this piecewise function is not smooth, though it is continuous, periodic, and symmetric. Namely, while cos *θ* is piecewise differentiable it is not differentiable at *t* = π/2,3π/2. A physical drop cannot take on such a nonsmooth shape^36,37,40−42^ on a smooth solid. One can consider drops that are templated to take a nonsmooth nonequilibrium shape^43^ and rough contacts may similarly cause a lack of smoothness, but this does not match the case presently considered.

Notice that the Furmidge-Kawasaki model is essentially a square wave, while the piecewise linear model is a triangular wave (which is a piecewise degree 1 polynomial). Interestingly, while the “zero-order” case of the Furmidge-Kawasaki model was discontinuous, the “first-order” piecewise linear case is discontinuous in its first derivative. As discussed further and shown in Section S4 of the Supporting Information, each model involving some *n*-degree piecewise polynomial has a discontinuity in its *n* th derivative. At the limit of an infinite series, the polynomial converges to the Taylor series of a sine wave, which is infinitely continuously differentiable. Now, in the case of a sine wave eq 5b is similar in form to the first term of a Fourier sine series.^44^ This resemblance to a Fourier series motivates the construction of such a series in modeling the general distribution of the contact angle along the triple line.

#### Fourier Series Model

In this section, we present a different approach to describing the Furmidge equation based on the description of the shape of the triple line, which, while still assuming some functional form of the contact angle distribution along the contact line, obeys physical criteria and agrees reasonably well with literature findings of *k* values and experimentally found contact line shapes.^15,36,38,45^ At the end of the preceding section, we noted that the piecewise polynomial model converges to a sine wave at infinite order. We hence describe cos θ(*t*) by a Fourier series with a period of 2π and only with terms that satisfy symmetry about the *y*-axis. We get
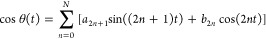
11

Where the *a*_1_ term must be nonzero to maintain a periodicity of 2π, and *N* can be a finite integer or infinite, provided it is not an infinite Fourier series that converges to a nonsmooth case such as those described in the previous section.^44^ More on this discussion can be found in Section S4 of the Supporting Information (see also^46^ therein). Noting the orthogonality of trigonometric functions, we obtain from eq 5b that
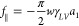
12a
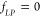
12bwhich automatically satisfies *f*_*LP*_ = 0. Additionally, eq 12a provides a physical perspective that *a*_1_ = 0 implies that *f*_∥_ = 0. Owing to the boundary conditions of *θ*_*A*_ and *θ_R_* at *t* = π/2 and, respectively, we obtain that
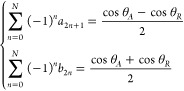
13

**Figure 3:**
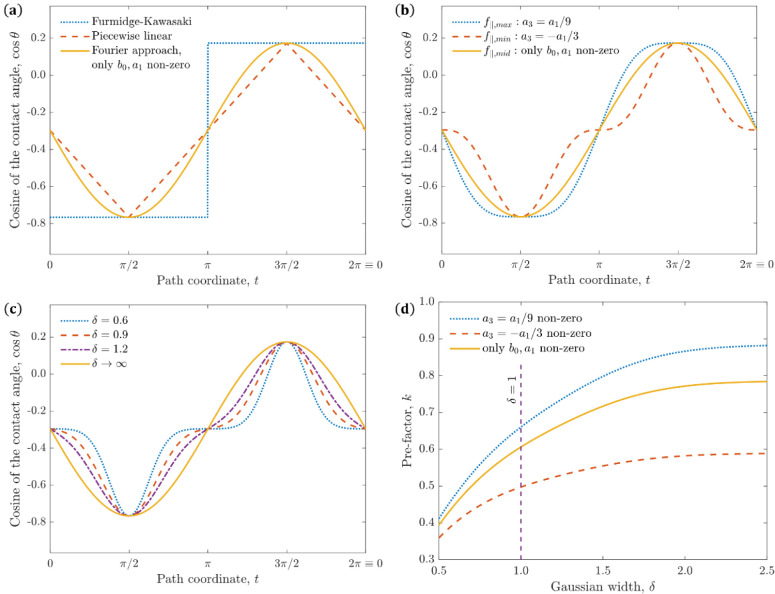
(a) The distribution of cos θ along the drop perimeter for different models, where for the sake of demonstration we take *θ*_*A*_ = 140°,*θ*_*R*_ = 80°. The inherently discontinuous model as well as linear model are both problematic due to discontinuity and the linear model also has asymmetry. On the other hand, the newly proposed model is both continuously periodic and symmetric, as is shown in the figure. (b) the distribution of cos θ along the drop perimeter for contact angle models corresponding to *f*_∥,*min*_,*f*_∥,*max*_,*f*_∥,*mid*_. We again take *θ*_*A*_ = 140°,*θ*_*R*_ = 80°. The minimum value of *k* corresponds to an inherent lingering on a value between the two angles, while the maximum value corresponds to a lingering on the two extreme values. (c) the variation of cos θ for different values of δ as presented in eq 16 compared to the base case of the Fourier model. We again take *θ*_*A*_ = 140°,*θ*_*R*_ = 80° as described above. We notice a gradual smoothing of the function between the limiting cases of *δ →* 0 and *δ →* ∞. Minimal and maximal Fourier cases of the Gaussian model are presented in Section S7 of the Supporting Information. (d) values of *k* plotted against δ ϵ (0.5,2.5). We see that a value of *k* = 0.5 is indeed reached, despite the previous findings of the Fourier series without the Gaussian extension.

Traditionally, given a Fourier series at two points means we only take two coefficients.^44^ In this base case, we take only *b*_0_,*a*_1_ nonzero and obtain the sine wave shown in Figure 3a alongside the models considered previously. We take sample values of *θ*_*A*_ = 140°, *θ*_*R*_ = 80° for the sake of the demonstration. The discontinuity of the Furmidge-Kawasaki model can be seen at the points *t* = π,2π. Additionally, the piecewise linear model’s nondifferentiability is observed at *t* = π/2,3π/2,0 ≡ 2π. Meanwhile, we see that the Fourier model base case provides continuity, periodicity, and symmetry about the *y* axis (see Figure 1) while remaining smooth throughout. Therefore, this contact model correctly captures the physical constraints of a sliding drop as it was intended to. Other sets of coefficients, of which there are infinite, will similarly satisfy the required constraints, while capturing additional physics.

We now expand on the base case by deliberately taking an arbitrary number of coefficients, noting that the base case balances physical accuracy and mathematical simplicity. In addition, we apply a condition that there be only two extrema at *θ*_*A*_ and *θ*_*R*_, as has been found experimentally for homogeneous surfaces.^15,16,36,45^ Since cos *θ* is monotonically decreasing in (0,π), we may equivalently state that there are only two extrema per period for cos *θ*(*t*), at cos *θ*_*A*_ and cos *θ*_*R*_. Consider the extrema obtained via the differentiation of cos *θ*:
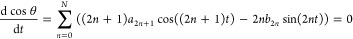
14

In general cases, such a series admits 2(2*N* + 1) roots per period, except when the trailing coefficients are all much smaller than *a*_1_.^47^ To determine the minimum and maximum values that *k* can take, we take only *b*_0_,*a*_1_,*a*_3_ to be nonzero and evaluate eq 14. Any additional *b*_2*n*_ terms added will not affect the value of *a*_1_ itself and only serve to increase the number of roots of eq 14, and any additional *a*_2*n*+1_ beyond *a*_3_ will increase the number of roots of eq 17 thereby allowing smaller variations of the value of *a*_1_. Namely, only *a*_3_ is added to the base case as it produces the smallest derivative in magnitude of the higher-order *a* terms as seen in eq 14, allowing the minimal/maximal values of *a*_1_ to be obtained. Plugging into eq 14 and requiring cos *t* not to have any roots in the domain (−1,1) yields that *a*_3_ must be between – *a*_1_/3 and *a*_1_/9. Further discussion on the above choices and calculations can be found in Section S5 of the Supporting Information. Combining the results in eqs 12 and 13 along with the range for *a*_3_, we find that
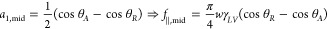
15a
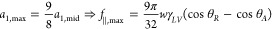
15b
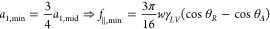
15cwhere *a*_1,*mid*_,*f*_∥,*mid*_ correspond to the base case and agree with the results of the preceding section, demonstrating the simplicity and accuracy of the base case. Meanwhile, *f*_∥_ can take any value between the minimal and maximal drop retention forces, *f*_∥,*min*_ and *f*_∥,*max*_. Note that *f*_∥,*min*_,*f*_∥,*max*_,*f*_∥,*mid*_ were obtained in the most general manner possible for a finite series. Therefore, they relay the properties that any Fourier series can take while satisfying the conditions prescribed to a physical sliding drop.

Recall that for a differential portion of the triple line the differential force is proportional to d*f* ∼ (cos *θ* - cos *θ*_*eq*_). In the case of a sliding drop, the drop naturally tilts in such a way that cos *θ*_*A*_ < cos *θ*_*eq*_ < cos *θ*_*R*_. The result of this is that the contact angle for a physical sliding drop must vary in some smooth and periodic manner while satisfying the conditions mentioned above. The physical significance of *f*_∥,*min*_,*f*_∥,*max*_ is therefore best explained by considering the variation of cos *θ* in each case, as shown Figure 3b. We see that *f*_∥,*min*_ is characterized by maximizing the part of the triple line which adopts contact angles close to an intermediate value between *θ*_*A*_ and *θ*_*R*_, and through a sharp rise to *θ*_*A*_ and fall to *θ*_*R*_, minimizes these parts near the advancing and receding edges. Such a drop contact line would appear slightly pinched but is still completely physical and infinitely continuously differentiable. On the other hand, *f*_∥,*max*_ corresponds to the opposite effect, namely that the majority of the advancing side takes on contact angles close to those of *θ*_*A*_ while the majority of the receding side takes on contact angles close to those of *θ*_*R*_, and there is a somewhat sharp change in the contact angle in the middle of the triple line, again maintaining infinite continuous differentiability.

**Figure 4:**
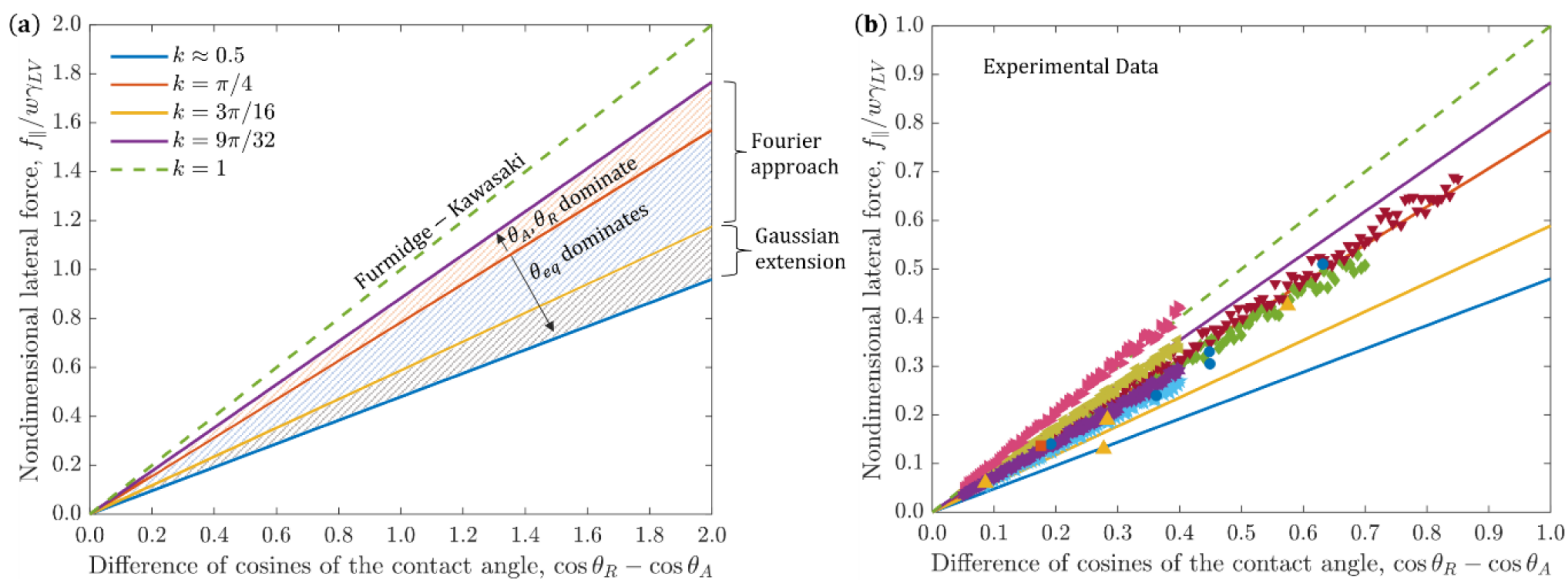
(a) nondimensional lateral parallel force, *f*_∥_/*w*γ*_LV_*, plotted against cos *θ*_*R*_ – cos *θ*_*A*_ for all physical models described up until now. The total range of *k* being between 0.5 and 0.9 is illustrated, and internal ranges are shown and described in terms of the effects of *θ*_*eq*_ against *θ*_*A*_,*θ*_*R*_ as well as the mathematical modeling. We also include a dashed line representing the Furmidge-Kawasaki model, which is out of range. (b) comparison to experimental data from,^13,16,18,20,48^ where data from^48^ are adapted from ACS Publishing (Copyright 2022 ACS Publishing), and data from^13,16,18,20^ are adapted with permission from Elsevier (Copyright 1962 Elsevier Publishing, Copyright 1990 Elsevier Publishing, Copyright 1995 Elsevier Publishing, Copyright 2008 Elsevier Publishing).

We see competition between two physical tendencies in a drop: the first is to remain near its equilibrium contact angle, and the second is to dichotomize to its advancing and receding values. The “outcome” of this competition depends on the solid–liquid interaction, and likely depends on physical parameters such as the interfacial modulus, interfacial tension, and equilibrium contact angle.^15^ Hence, we describe values of *k* below *f*_∥,*mid*_ as *θ*_*eq*_-dominated, and values of *k* above *f*_∥,*mid*_ as *θ*_*A*_,*θ*_*R*_-dominated with regards to the outcome of the above competition. Weaker forces pertain to *θ*_*eq*_-dominated systems, and vice versa, as observed in Figure 4b (Section S3 of the Supporing Information), where more experimental points are observed in the *θ*_*A*_,*θ*_*R*_-dominated region. Note that the inclination angle at which the drop is sliding depends implicitly on the above physical parameters, and in being the reason for the introduction of asymmetry in the system in the first place, is a parameter through which the outcome of the above competition could be inferred.

#### Gaussian Extension

So far, we have found that *k* can take values between 3π/16 and 9π/32. Nevertheless, the experimental values of *k* found in the literature have a much wider range. We now extend our Fourier model to accommodate values below our current range. In a manner opposite to, and equally unphysical as the Furmidge-Kawasaki model, consider the Dirac delta distribution as the contact angle variation. A superposition of Dirac deltas about *θ*_*A*_ and *θ*_*R*_ yields *k* = 0, a clearly unphysical yet fascinating result. In such a case, the drop is essentially at a constant intermediate value everywhere except its edges. While the Furmidge-Kawasaki model is discontinuous about the middle of the drop, this model would be discontinuous at the edges. Now, a superposition of Gaussian curves of width *δ* about *θ*_*A*_ and *θ*_*R*_ presents a possibility for a correction of the Dirac deltas, constructing a drop whose contact angle varies smoothly and symmetrically. To ensure periodicity and symmetry are met, we multiply the Gaussian terms by our Fourier series (except for the free term) and superpose the Gaussians on the entirety of the real number line, producing the desired infinitely periodic behavior. This way, the “tails” of the Gaussians, which extend infinitely, can cancel each other out in a symmetric manner so that symmetry and periodicity are enforced. Finally, to meet the BCs we renormalize the function. See Section S6 of the Supporting Information for a more detailed walkthrough of this procedure. Applying this on the base case of the Fourier series yields
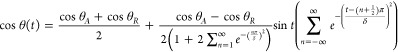
16

We recognize two limiting cases: in the case where *δ* → 0 we get back the unphysical Dirac delta distribution, whereas by tending *δ* → ∞ we get back the base case of the Fourier model without the Gaussian extension. We therefore recognize that we can indeed multiply our Fourier series with an infinite series of superposed Gaussians (Gaussian series) to obtain a new function form for modeling cos *θ* (*t*). While the significance of *δ* is mainly mathematical, its physical perspectives is related to the portion of the contact line at which the drop is more drawn to the advancing and receding contact angles than to its equilibrium contact angle. Figure 3c illustrates some possibilities for the variation of the contact angle given different values of *δ*. Based on this figure, we deduce that the minimal physical value of *δ* is between 0.9 and 1.2. This allows us to expand upon the physics described above and introduces another physical parameter which is dependent on the solid–liquid contact and therefore dictates *k*.

By combining the model for the base case in eq 16 with the relation from eq 5b, we obtain
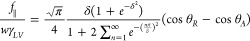
17

eq 17 is then expanded to the minimal and maximal Fourier cases (see Section S6 of the Supporting Information for the mathematical expressions), and all three are plotted in Figure 3d against *δ*. We see that for larger values of *δ*, *k* plateaus to the values corresponding to the Fourier model without the Gaussian extension. The minimal curve yields *k* ≈ 0.5 at *δ* ≈ 1, representing the lower bound of experimental results while supporting the minimal value of *δ* being between 0.9 and 1.2.^15^

## Results and Discussion

At this point, our model covers all bases in terms of possible *k* values other than those approaching or greater than 1 (which will be reconciled in the following section). We have shown that the lower bound of *k* is somewhere slightly below 0.5, and that the upper bound is 9π/32, slightly below 0.9, as shown in the master plot Figure 4. As mentioned above, the physical significance of this range is the competition between the tendency of the drop to remain near its equilibrium contact angle and the tendency to dichotomize to its advancing and receding values. The base case of the Fourier series presents the middle ground, where the transition from the two values is steady. In cases where an intermediate position is preferred, we get a lower lateral parallel force as the drop is overall closer to its equilibrium contact angle throughout. Meanwhile, when the two regions dichotomize the drop is overall further from equilibrium and therefore the lateral parallel force is higher.

Based on the above results, we indeed observe a range of *k* values for different solid–liquid systems pertaining to different “outcomes” of the competition between the two tendencies of the drop described above. Essentially, in view of the wide spectrum obtained, each set of results can be identified with a different contact angle distribution from those which we have described above. In addition, notable application of our modeling and results is therefore as follows: by measuring the distribution of contact angles at the time of sliding one can fit their results to one of the contact angle distribution models presented here and obtain a value for *k* without the need to interfere with the drop’s motion to measure its force directly.

Finally, the value *k* = 1 comes up frequently in the experimental data. we have calculated the drop retention forces by only taking into account the solid–liquid interaction at the triple line. We did this because often *f*_∥_ is taken as a boundary condition at the triple line in numerical drop dynamics simulations.^49−57^ However, if one is considering the total frictional force in a sliding drop experiment, one must also consider the forces at the solid–liquid surface. Note that the viscous dissipation of the molecules within the liquid drop, which is important for energetic considerations, is expressed force-wise at the solid–liquid interface only. As we consider slow sliding, the creeping flow behavior of the bulk, as well as the solid–liquid interface (excluding the contact line), is dominated by viscous drag. As a result, we can write that, more generally, the lateral parallel force is composed of a triple-line contribution and a surface contribution, i.e.
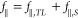
18where *f*_∥,*TL*_ and *f*_∥,*S*_ represent the frictional force at the triple line and at the solid–liquid surface, respectively. Therefore, it is likely that in such studies where *k* ≈ 1, the viscous contribution is significant and the triple line contribution is not the only force being measured (though it is still the more significant force). These are likely to be larger drops, as discussed via scaling in Section S8 of the Supporing Information (see also^15,58−63^). As recent experimental work has shown that the viscous contribution does not affect *θ*_*A*_ or *θ*_*R*_,^64^ we can safely delineate *f*_∥,*TL*_ and *f*_∥,*S*_, taking them to be independent.

### Fitting the Contact Angle Variation to Experimental Data

**Figure 5:**
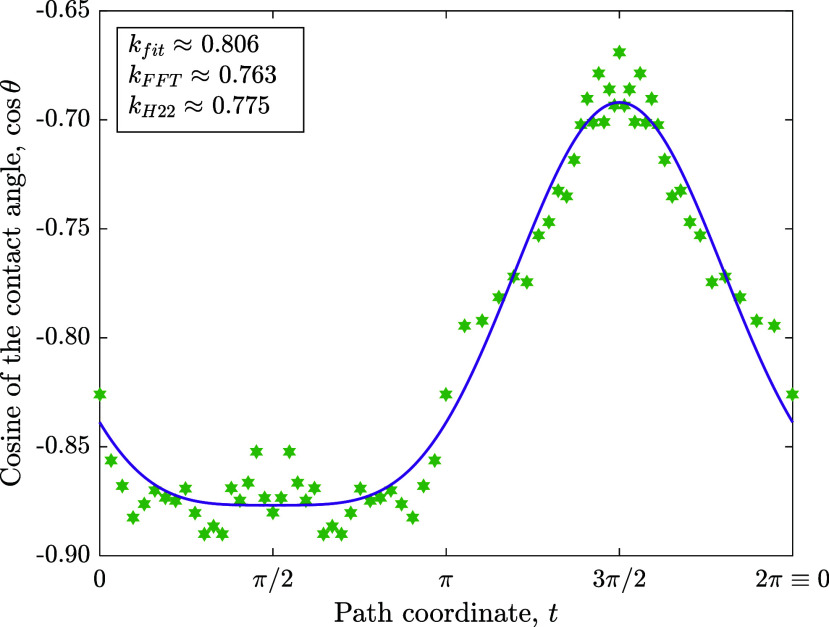
Fitting the Fourier model (with *b*_0_,*a*_1_,*b*_2_,*a*_3_ nonzero) with data adapted from^48^ (Copyright 2022 ACS Publishing). We observe good agreement between the fit and the data, as well as between the *k* values obtained (both by direct fitting and FFT) to the *k* value obtained in the reference.

We use available experimental results obtained in^48^ for a case of *θ*_*A*_ = 152° and *θ*_*R*_ = 135°. Our fit, as shown in Figure 5 below, exhibits good agreement between a Fourier model (with *b*_0_,*a*_1_,*b*_2_,*a*_3_ nonzero) and the data. There are two ways to compute the value of *k* from the data: the first is to compute it directly from the fitted value of *a*_1_, and the other is to perform a fast-Fourier transform (FFT) on the data. We denote these values as *k*_*fit*_ and *k*_*FFT*_, respectively. As shown on Figure 5, there is a 5% relative difference between *k*_*fit*_ and *k*_*FFT*_, and they are both within a 4% relative difference from the value obtained in the reference (*k*_*H*22_ in Figure 5). Moreover, the fit itself has a root-mean square error of 1.2% with R^2^ ≈ 98%, while the experimental measurements themselves are reported to have an uncertainty of ± 2°.^48^ Hence, the discrepancies can be interpreted as experimental measurement errors, and the data support the model put forth here.

Interestingly, it can be seen in Figure 5 that the largest disparities between the fit and the experimental data occur at *t* = 3π/2 and at *t* = π/2. As these positions indicate the receding and advancing edges of the drop, it is possible that the optics regarding the elliptical drop introduced a slight difficulty in the measurement at those points, causing larger errors, hence the larger disparity. Curiously, in both cases, the error is in the same direction.

## Conclusions

We derive the very first model that yields a range of Furmidge-corrective *k* values, as opposed to single values proposed by other authors.^13,15,16,19^ Our range, that largely lies between 0.5 and 0.9, can be divided into two main regions: one dominated by the tendency of a drop to adopt contact angles near the equilibrium angle *θ*_*eq*_, and another dominated by the opposite tendency of a drop to adopt contact angles near the advancing and receding values *θ*_*A*_,*θ*_*R*_. By comparing the *k* values obtained by our model to those obtained in the literature, we affiliate higher retention force systems with the *θ*_*A*_,*θ*_*R*_-dominated region, while lower retention force systems span the entire range and also above it. We reconcile *k* values falling above our range by considering the effect of viscous drag forces during sliding. Further to this, we use our general model to fit experimental contact angle distribution data and find good agreement with both the data and the *k* value arising from it.

Moreover, our model for the distribution of the contact angle is physical: while previous models are discontinuous (or have some discontinuous derivatives),^13,15−19^ our model is continuously differentiable for all orders, is periodic and symmetric with respect to the *y* axis, and only has the only two required extrema at the advancing and receding ends of the drop. These properties are obtained via a Fourier series model of the contact angle and expanded on by multiplying the Fourier series with a Gaussian series to obtain the lower values in our range. Namely, as shown in Figure 4a, the upper part of our obtained range, with *k* values between 3π/16 and 9π/32, is obtained via the Fourier model, while lower values down to *k* ≈ 0.5 are admitted via the Gaussian extension, and there is a significant gap between our upper bound and the original Furmidge model for which *k* = 1.

The significance of the analysis presented here is 2-fold. First, the understanding of the physical constraints on a sliding drop is crucial to the modeling of drops in various simulations.^49−57^ Moreover, our results help clarify the physics behind sliding drops as well as the applicability of the Furmidge eq. In cases where just the triple line forces need to be considered, the prefactor *k* ranges between 0.5 and 0.9. In other cases, the effects of viscosity must be taken into account. In other cases still, surfaces and drops can be templated or engineered to break the physical constraints imposed here to obtain values of *k* outside the normal range, and even to obtain a *k* value that changes in time providing the system is designed so that the drop continues sliding steadily.^15,26,53,57,65,66^ Any of these approaches as well as a combination of them may be used as boundary conditions or design equations in a plethora of scientific and engineering fields dealing with sliding drops.^1−12^

Ultimately, we explain analytically the wide variety of experimental results as well as provide both a physical interpretation as well as methods of identification and application. While this work further advances our understanding of the contact angle distribution as well as contact line forces on sliding drops, it also underscores that an in-depth understanding of the dynamics involved remains lacking, and that further investigations are needed. Future studies should aim to expand on current experimental results as well as expand on this model for rough surfaces, complex fluids, and nonelliptical contacts. Particularly, the case of rough or textured surfaces (including superhydrophobic surfaces) poses a fascinating challenge,^67−70^ as the variation of the contact angle along the triple line would be subject to the microscopic peaks and troughs on the surface. Therefore, the challenge in such a case would be to develop a mathematical model that could account for these variations, and, most likely, a numerical framework would be needed to analyze each different rough/textured case. Furthermore, the consequences of breaking some of the imposed physical constraints as outlined in this paper should be studied, where one may expect to show that in certain cases, values of *k* > 1 can be achieved (e.g., a patterned surface). In addition, the exact effect of viscous drag should be investigated in depth. Further studies could also use tools such as image processing with deep learning in conjunction with the general model presented here in order to model a wider range of drops and surfaces.^71^ Finally, microscopic-based studies, especially those dealing with molecular dynamics simulations could shed more light on wetting, pinning-depinning,^67^ and forces as a whole in the context of sliding drops,^26,38,72^ dealing with, for example, the effect of microstructure on the contact angle variation.^64,68^ The results from this study in conjunction with any of those mentioned should open the door toward a near-universal model of sliding drops to a wide variety of scientific and engineering fields.

## Supporting Information Available

Further derivations and theoretical details; S1. Drop in equilibrium over a smooth, flat surface; S2. Two-ellipse contact geometry; S3. Cusp geometry; S4. Discontinuity/nondifferentiability and more piecewise polynomial models; S5. Minimal and maximal Fourier cases; S6. Basis for the Gaussian model and deviation from BCs; S7. Minimal and maximal Fourier cases with Gaussian extension; S8. Comparison of *f*_∥,*TL*_ and *f*_∥,*S*_ (PDF)
