# Supplementary material for: Furmidge Equation Revisited
Source: Langmuir. 2025 May 2;41(18):11785–93. doi: 10.1021/acs.langmuir.5c01302 (PMC12080332; doi:10.1021/acs.langmuir.5c01302)
Supplement: Supplementary file 1 — la5c01302_si_001.pdf [file la5c01302_si_001.pdf]

Furmidge equation revisited

Supplementary Material

Yotam Stern [a,*], Rafael Tadmor [a], Assaf Miron [a], Appu Vinod [a]

[a] *Dept. of Mechanical Engineering, Ben Gurion University of the Negev, Beer Sheva, Israel. Postal code 8410501*

*Corresponding author.

E-mail address: sternyo@post.bgu.ac.il (Y. Stern).

Table of Contents

S1. Drop in equilibrium on a smooth, flat surface 2

S2. Two semi-ellipse contact geometry 2

S3. Cusp geometry 3

S4. Discontinuity/nondifferentiability and more piecewise polynomial models 5

S5. Minimal and maximal Fourier cases 8

S6. Basis for the Gaussian model and deviation from BCs 9

S7. Minimal and maximal Fourier cases with Gaussian extension 10

S8. Comparison of $f_{\parallel,TL}$ and $f_{\parallel,S}$ 11

S1. Drop in equilibrium on a smooth, flat surface

Below, we show a schematic of drop in equilibrium on a smooth, flat surface which has a circular contact with the surface:

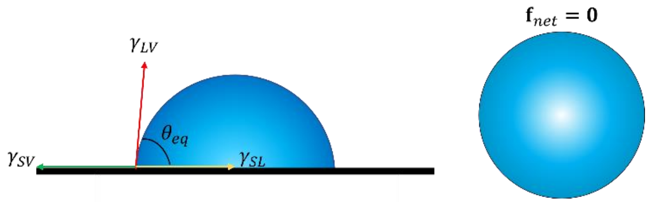

Fig. S1. Schematic of a drop at rest on a flat solid surface. The net force acting on the drop is zero, owing to a force balance between the solid-vapor, solid-liquid, and liquid-vapor surface tensions $\gamma_{SV}, \gamma_{SL}, \gamma_{LV}$ at a given equilibrium contact angle θ_{eq}.

As shown above, this equilibrium is what results in the Young equation,

$$\gamma_{SV} - \gamma_{SL} - \gamma_{LV} \cos\theta_{eq} = 0\,, \tag{S1}$$

where $\gamma_{SV}, \gamma_{SL}, \gamma_{LV}$ are the solid-vapor, solid-liquid, and liquid-vapor surface tensions and θ_{eq} represents the equilibrium contact angle for the drop at rest.

S2. Two semi-ellipse contact geometry

As shown below in **Fig. S2**, the parameterization in the case of a two semi-ellipse contact model is comprised of $t_{top} \in (0, \pi)$ and $t_{bottom} \in (\pi, 2\pi)$, while both parameterizations share a common value for $a_{top} = a_{bottom}$ and different values of $b_{top} \neq b_{bottom}$. Plugging this into Eq. 6 of the main paper yields

$$f_{\parallel} = -a\gamma_{LV} \int_0^{2\pi} \cos\theta \sin t \, \mathrm{d}t\,, \tag{S2a}$$

$$f_{LP} = -\gamma_{LV} \left(b_{top} \int_0^{\pi} \cos\theta \cos t \, \mathrm{d}t + b_{bottom} \int_{\pi}^{2\pi} \cos\theta \cos t \, \mathrm{d}t \right) = 0\,. \tag{S2b}$$

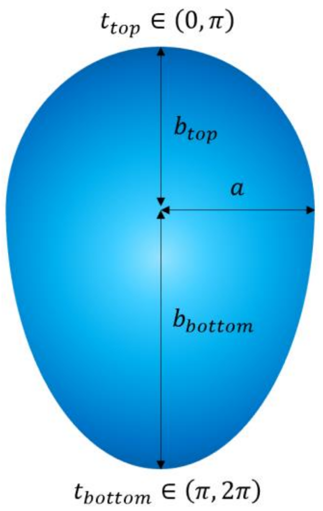

Fig. S2. Two-ellipse contact model, where $t_{top} \in (0,\pi), t_{bottom} \in (\pi, 2\pi)$. By continuity $a_{top} = a_{bottom}$, while by the definition of the model $b_{top} \neq b_{bottom}$. All the properties of symmetry and periodicity are conserved, therefore the same results are obtained compared to a single ellipse contact model.

Hence, we have shown that the results obtained using simple elliptical contact geometry will yield the same results as this contact geometry. The importance of this result lies in our ability to accurately capture the physics of a sliding drop in as simple a manner as possible.

S3. Cusp geometry

As mentioned in the main paper, some studies have found that the contact geometry of sliding drops often contains a cusp as shown in **Fig. S3** [S1–S4]. In such cases, we may still use elliptical parameterization for most of the drop, and for some region around $t = 3\pi/2$ the contact geometry is assumed to be triangular/linear. Then, we have that for the region around cusp

$$\hat{n}_y \mathrm{d}s = -\mathrm{d}x \,, \tag{S3a}$$

$$\hat{n}_x \mathrm{d}s = \mathrm{d}y \,. \tag{S3b}$$

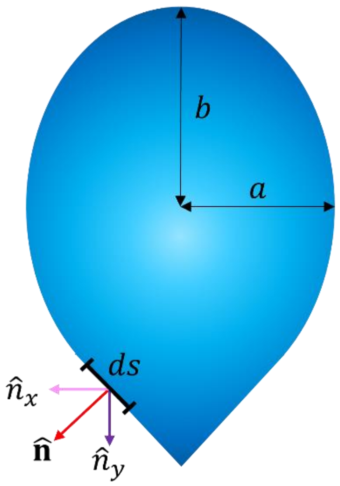

$$t_{cusp} \in \left(\frac{3\pi - \varphi}{2}, \frac{3\pi + \varphi}{2}\right)$$

Fig. S3. Contact geometry containing a cusp where $t_{cusp} \in ([3\pi - \varphi]/2, [3\pi + \varphi]/2)$. The normal vector and its components as well as a sector length are shown for the cusp region.

Now, invoking symmetry while denoting φ the parameterization length of the cusp, we obtain

$$f_{\parallel} = -2\gamma_{LV}\left(a\int_{\frac{\pi}{2}}^{\frac{3\pi-\varphi}{2}} \cos\theta \sin t \, \mathrm{d}t - \int_{a\cos\frac{3\pi-\varphi}{2}}^{0} \cos\theta \, \mathrm{d}x\right), \tag{S4a}$$

$$f_{LP} = 0\,, \tag{S4b}$$

where, as before we have that $f_{\parallel} \neq 0$ and $f_{LP} = 0$ by symmetry. Taking the base case of the Fourier approach, while extending $\cos\theta = \sin t$ to the cusp region, where $x = a\cos t$ and therefore $t = \arccos(x/a)$, we get that

$$f_{\parallel} = \frac{1}{4} w\gamma_{LV}(\cos\theta_R - \cos\theta_A)(\pi - \varphi - \sin\varphi)\,, \tag{S5}$$

which reduces to the same solution found in Eq. 18a of the main paper for $\varphi = 0$, and then decreases monotonically $\forall\varphi \in [0, \pi]$. This monotonic decrease is shown in **Fig. S4**, where we compare the pre-factor k to the cusp parameterization length φ. We see that indeed the pre-factor decreases monotonically and in fact reaches the experimentally-found value of $k = 0.5$ at $\varphi \approx 0.6$. In other words, in the case of a small drop for which $\mathrm{Bo} \ll 1$ and for which a significant cusp forms, the lower experimental values from the literature are obtained even

without the minimal Fourier case or Gaussian extensions. It is also possible to reach these values for Bo $\lesssim 1$ and for $\varphi \approx 1$, which represents a large cusp but one may still be physical. Typically, the formation of a cusp occurs in part due to viscous effects, therefore this occurrence is logical especially if compounded to a solid-liquid contact that lends itself to the aforementioned minimal Fourier case or Gaussian extension. This result therefore strengthens those in the main paper that show that $k < 1$ even if k_{eff} may be greater than 1.

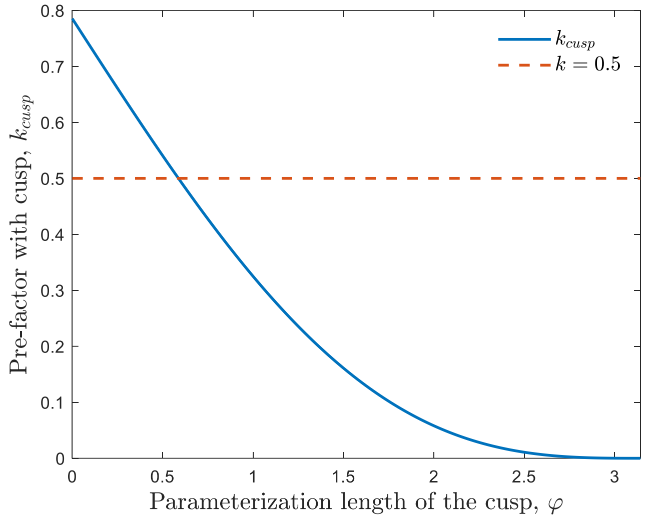

Fig. S4. Values of k for the base case of the Fourier approach plotted against different cusp lengths φ. It can be seen that a cusp reduces the value of k, strengthening the results of the main paper.

S4. Discontinuity/nondifferentiability and more piecewise polynomial models

For the "zero-order" polynomial case, we define the magnitude of the discontinuity as the difference between the opposing limits of $\cos\theta$ at the point of discontinuity, which, for the Furmidge model is

$$\Delta_0 = \cos\theta_R - \cos\theta_A \ . \tag{S6a}$$

Meanwhile, for the piecewise linear model, the magnitude of the nondifferentiability (defined analogously to the magnitude of discontinuity) is

$$\Delta_1 = \frac{2}{\pi}(\cos\theta_R - \cos\theta_A) \ . \tag{S6b}$$

We notice a similarity between the discontinuity of the Furmidge-Kawasaki model and the nondifferentiability of the piecewise continuous model. Notice that the Furmidge-Kawasaki

model is essentially a square wave, while the piecewise linear model is a triangular wave (which is a piecewise degree 1 polynomial). Such discontinuity or non-differentiability would impact simulations, as the ability of the numerical framework to deal with the discontinuity at the contact line would be more limited. A simulation requires the calculation of numerical derivatives of at least the second order (owing to the divergence of the stress tensor, $\boldsymbol{\nabla} \cdot \boldsymbol{\tau}$, assuming Newtonian flow), such that a discontinuity in the function or in its first derivative would be expected to diffuse into the bulk behavior in the simulation. In fact, a discontinuity of any derivative of the drop shape may have an impact on the dynamics in a simulation.

Further to the above, we wish to generalize this for a piecewise polynomial of degree n, expecting to arrive at

$$\Delta_n = k_n(\cos\theta_R - \cos\theta_A) = \frac{f_{\parallel n}}{w\gamma_{LV}} , \tag{S6c}$$

where Δ_n represents a discontinuity in the nth derivative. While they are solvable analytically, the piecewise polynomial models between 2nd and 6th order were solved symbolically in MATLAB owing to convenience. In each case, the boundary conditions as well as conditions for symmetry and smoothness up to $n-1$ order were collocated into a matrix which was solved symbolically. The result was integrated symbolically according to Eq. 6 in the main paper, and the result was converted to double. Finally, the coefficients of each section of each piecewise polynomial were compared and the following results were obtained:

$$\begin{cases} n = 2 & k_2 = \dfrac{8}{\pi^2} & \Delta_2 = \dfrac{8}{\pi^2}(\cos\theta_R - \cos\theta_A) \\ n = 3 & k_3 = \dfrac{24}{\pi^3} & \Delta_3 = \dfrac{24}{\pi^3}(\cos\theta_R - \cos\theta_A) \\ n = 4 & k_4 = \dfrac{76.8}{\pi^4} & \Delta_4 = \dfrac{76.8}{\pi^4}(\cos\theta_R - \cos\theta_A) \\ n = 5 & k_5 = \dfrac{240}{\pi^5} & \Delta_5 = \dfrac{240}{\pi^5}(\cos\theta_R - \cos\theta_A) \\ n = 6 & k_6 = \dfrac{755.4097}{\pi^6} & \Delta_6 = \dfrac{755.4097}{\pi^6}(\cos\theta_R - \cos\theta_A) \end{cases} . \tag{S7}$$

These results indeed provide evidence that $\Delta_n = k_n(\cos\theta_R - \cos\theta_A)$, as conjectured. They also show that k approaches $\pi/4$, which corresponds to the fact that an infinite polynomial

series would converge to the base case of the Fourier approach (i.e., a sine wave) as mentioned and explained in the main paper.

In the case of an infinite series, we converge at the Taylor series of a sine wave, with a coefficient of $a_1 = (\cos\theta_A - \cos\theta_R)/2$. In this case, as mentioned in the main paper, we notice that t and $\sin t$ are both real-valued, hence $\sin t$ is not analytic in the complex plane. The measure of this non-analytic behavior is given by $|1/2 \cdot \partial/\partial t([\cos\theta_A - \cos\theta_R]/2 \cdot \sin t)| = (\cos\theta_R - \cos\theta_A)/4 \cdot \cos t$. Since the function $\cos t$ takes different values in the domain, we take its second moment as given by

$$\langle \cos t \rangle^2 = \int_0^{2\pi} \cos^2 t \, \mathrm{d}t = \pi \, . \quad \text{(S8)}$$

Hence, we indeed obtain that

$$\Delta_\infty = \frac{\pi}{4}(\cos\theta_R - \cos\theta_A) = k_n(\cos\theta_R - \cos\theta_A) = \frac{f_{\parallel\infty}}{w\gamma_{LV}} \, , \quad \text{(S9)}$$

as posited in the main paper.

One can, in fact, calculate the value of k for all piecewise polynomial models by considering infinite Fourier series. This is in fact the logic that restricts the Fourier approach in the main paper to finite series. For example, the square wave is given by the infinite Fourier series[5]

$$\cos\theta = \frac{\cos\theta_A + \cos\theta_R}{2} + \frac{2}{\pi}(\cos\theta_A - \cos\theta_R) \sum_{m=1}^{\infty} \frac{\sin([2m-1]t)}{2m-1} \, . \quad \text{(S10)}$$

Due to the discontinuity, this infinite Fourier series is subject to Gibbs phenomenon, where a lot of noise appears near the discontinuity[5]. Now, one can take the antiderivative of the series portion of this function to obtain the triangular wave, however we need to change the pre-factor of the infinite series term to satisfy boundary conditions[5]. Upon doing so and applying some trigonometric manipulation, we arrive at

$$\cos\theta = \frac{\cos\theta_A + \cos\theta_R}{2} - \frac{4}{\pi^2}(\cos\theta_A - \cos\theta_R) \sum_{m=1}^{\infty} \frac{(-1)^m \sin([2m-1]t)}{(2m-1)^2} \, . \quad \text{(S11)}$$

A pattern can be seen to be emerging. In the square wave case we have divided by two and also by $\pi/4$, while in the triangular wave case we have divided by two and by $\pi/4$, then multiplied by $2/\pi$. We can hence write the following general formula for a piecewise polynomial of degree n:

$$\cos\theta = \frac{\cos\theta_A + \cos\theta_R}{2} + \frac{2}{\pi} k_n (\cos\theta_A - \cos\theta_R) \sum_{m=1}^{\infty} \frac{(-1)^{n(m+1)} \sin([2m-1]t)}{(2m-1)^{n+1}} \ . \quad \text{(S12)}$$

Because $\cos\theta\,|_{t=\pi/2} = \cos\theta_A$, we get the following formula for k_n:

$$k_n = \frac{\pi}{4} \left(\sum_{m=1}^{\infty} \frac{[-1]^{n[m+1]} \sin\left[\{2m-1\}\frac{\pi}{2}\right]}{[2m-1]^{n+1}} \right)^{-1} \ . \quad \text{(S13)}$$

This allows us to calculate k_n for any polynomial, as shown in **Fig. S5**. Notice also that when $n \to \infty$, we indeed return to the base Fourier case of a simple sine wave, with $k = \pi/4$[5].

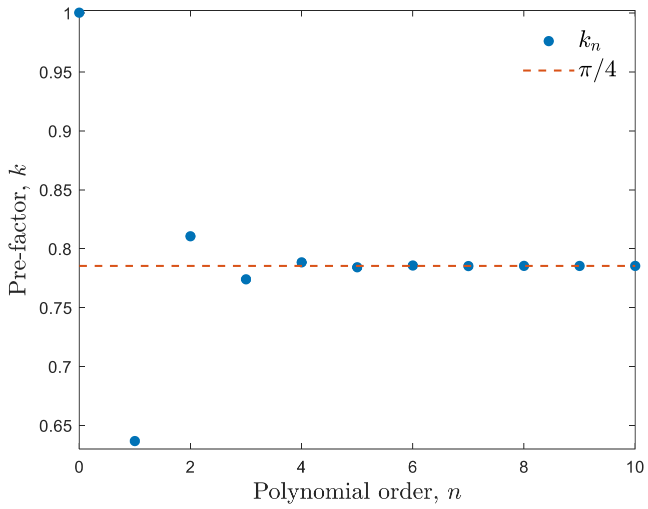

Fig. S5. Values of k for piecewise polynomials of degree n. We see a convergence towards $\pi/4$ as $n \to \infty$.

S5. Minimal and maximal Fourier cases

As discussed in the main paper, only the terms b_0, a_1, a_3 are taken in order to identify the minimal and maximal values that a_1, and therefore k can take under the Fourier series model. As discussed in the main text, b_0 will not influence this value, and therefore we must find the

range of a_3. This is obtained by plugging in and obtaining the functional form $a_1 \cos t + a_3 \cos 3t$, which can be equivalently written as $a_1 \cos t + a_3(4\cos^3 t - 3\cos t)$. Substituting $X \triangleq \cos t$, we get

$$X + \frac{a_3}{a_1} 3X(4X^2 - 3) = 0 \,. \tag{S14a}$$

Recall that $X = 0$ implies $t = \pi/2$,$3\pi/2$ as expected. For roots $X \neq 0$ which we want to avoid, we obtain

$$1 + \frac{a_3}{a_1} 3(4X^2 - 3) = 0 \,, \tag{S14b}$$

so that

$$X = \pm\sqrt{\frac{3}{4} - \frac{1}{12a_3/a_1}} \,. \tag{S14b}$$

Therefore, the first possible case is $X \notin \mathbb{R}$, for which $a_3/a_1 \leq 1/9$. The second case is $|X| > 1$, as $|\cos t| > 1$ implies that $t \notin \mathbb{R}$. This results in $a_3/a_1 \geq -1/3$. Therefore, the range of $a_3 \in (-a_1/3, a_1/9)$ is obtained.

S6. Basis for the Gaussian model and deviation from BCs

The Gaussian model in its original form is written as

$$\cos\theta\,(t) = \frac{\cos\theta_A + \cos\theta_R}{2} + \frac{\cos\theta_A - \cos\theta_R}{2}\left(e^{-\left(\frac{t-\frac{\pi}{2}}{\delta}\right)^2} - e^{-\left(\frac{t-\frac{3\pi}{2}}{\delta}\right)^2}\right), \tag{S15}$$

and it is seen that when $t = \pi/2$, we get a slight deviation of

$$\cos\theta\left(\frac{\pi}{2}\right) = \cos\theta_A + \frac{\cos\theta_R - \cos\theta_A}{2} e^{-\left(\frac{\pi}{\delta}\right)^2} \,, \tag{S16}$$

which shows that boundary conditions are not satisfied. By summing up Gaussians from $-\infty$ to ∞ and multiplying by the non-constant terms of the Fourier series, we guarantee that boundary conditions as well as the rest of the physical properties of symmetry and periodicity are satisfied. Furthermore, renormalizing and dividing by a factor of $2\left(1+2\sum_{n=1}^{\infty} e^{-\left(\frac{n\pi}{\delta}\right)^2}\right)$ allows for a correction that works $\forall\delta$.

S7. Minimal and maximal Fourier cases with Gaussian extension

Similar to the base case, we plotted $\cos\theta\,(t)$ at different values of δ. Similarly to what was discussed in the main paper, lower values of δ below $\delta \approx 0.9$ are deemed unphysical while in the limit of $\delta \to \infty$ we revert back to the Fourier approach without the Gaussian extension.

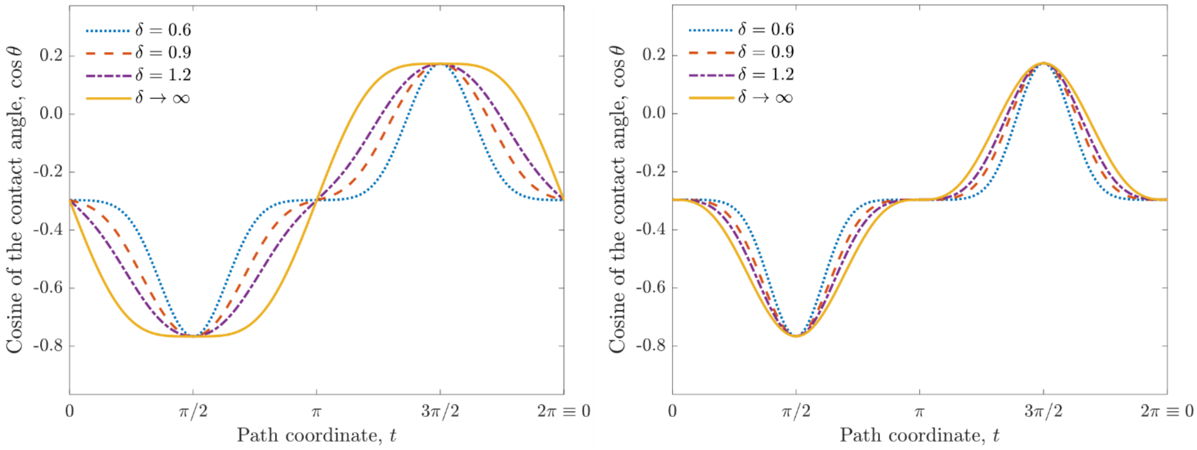

Fig. S6. Plots of the maximal (left) and minimal (right) Fourier approaches with the Gaussian series extension. It can be seen that in both cases high values of $\delta \to \infty$ revert back to the Fourier case without the Gaussian extension. Lower values of δ seem somewhat unphysical, as discussed in the main paper.

Now, in terms of the lateral parallel force the minimal and maximal Fourier cases provide slightly different results from that of the base case. Namely, for these cases we get

$$\frac{f_{\parallel,max}}{w\gamma_{LV}} = \frac{\sqrt{\pi}}{32}\frac{\delta\left(9+8e^{-\delta^2}-e^{-4\delta^2}\right)}{1+2\sum_{n=1}^{\infty} e^{-\left(\frac{n\pi}{\delta}\right)^2}}(\cos\theta_R - \cos\theta_A)\,, \tag{S17a}$$

$$\frac{f_{\parallel,min}}{w\gamma_{LV}} = \frac{\sqrt{\pi}}{16}\frac{\delta\left(3+4e^{-\delta^2}+e^{-4\delta^2}\right)}{1+2\sum_{n=1}^{\infty} e^{-\left(\frac{n\pi}{\delta}\right)^2}}(\cos\theta_R - \cos\theta_A)\,. \tag{S17b}$$

These results combine with Eq. 20 of the main paper to give Fig. 8 of the main paper. Note that in both cases, as $\delta \to \infty$ we get back values of $k = 9\pi/32, 3\pi/16$, corresponding respectively to the maximal and minimal values of the Fourier approach without the Gaussian extension.

S8. Comparison of $f_{\parallel,TL}$ and $f_{\parallel,S}$

As mentioned in the main paper, the total force on a sliding droplet includes the triple line force, $f_{\parallel,TL}$, and some force at the solid-liquid surface, $f_{\parallel,S}$, which has to do with viscous drag. This is shown in **Fig. S7** below:

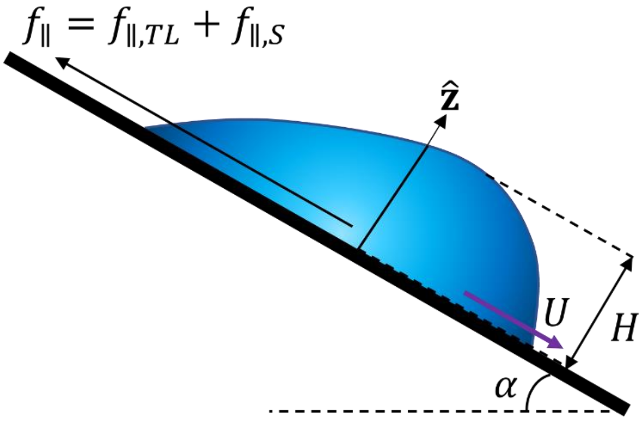

Fig. S7. The surface shear force as a result of the motion of the drop, which scales linearly with dynamic viscosity, velocity of motion, and scales to the area of the solid-liquid interface divided by the height of the drop.

The surface force is the sum of the surface stresses[6], hence we write that

$$f_{\parallel,S} = \int_{A_{SL}} \mu \frac{\partial u}{\partial z}|_{z=0}\, dA_{SL} \sim \mu \frac{U}{H} ab\,, \qquad \text{(S18a)}$$

$$f_{\parallel,TL} = kw\gamma_{LV}(\cos\theta_R - \cos\theta_A) \sim a\gamma_{LV}\,, \qquad \text{(S18b)}$$

where A_{SL} represents the solid-liquid contact area, μ is the dynamic viscosity of the fluid, $u(z)$ is the fluid velocity in the parallel depending on the vertical coordinate z, H is the height of the drop, U represents the velocity at which the drop is sliding, and a and b are the semi-major and semi-minor axes as defined by Eq. 2 in the main paper. Notice that when the drop is static, i.e. $U = 0$, $f_{\parallel,S}$ is identically zero. This presents a quintessential difference between the frictional

behavior of solids and liquids: while in solids the dynamic friction is typically lower than the static friction, in liquids friction during motion is higher because the flow of the liquid itself adds internal friction to that of the triple line forces. Rather, the relation between static and dynamic friction in solids is better analogized to the relation between the depinning force and $f_{\parallel,TL}$ in liquids, as discussed in [7–11].

Reverting to the analysis of $f_{\parallel,S}$, we can compare the two forces defined by Eq. S18:

$$\frac{f_{\parallel,S}}{f_{\parallel,TL}} \sim \frac{\mu \frac{U}{H} ab}{a\gamma_{LV}} \sim \frac{b}{H}\frac{\mu U}{\gamma_{LV}} \,. \tag{S19}$$

Essentially, we have obtained the capillary number, Ca $\triangleq \mu U/\gamma_{LV}$, multiplied by a geometrical factor of b/H. However, we do not know the velocity of the drop a priori. Writing momentum balances for the drop in the $\hat{\mathbf{y}}, \hat{\mathbf{z}}$ directions, assuming Stokes flow within the drop[12], we attain that

$$-\frac{\partial p}{\partial y} + \mu \frac{\partial^2 u}{\partial z^2} - \rho g \sin\alpha = 0 \,, \tag{S20a}$$

$$-\frac{\partial p}{\partial z} - \rho g \cos\alpha = 0 \,, \tag{S20b}$$

where α is the angle of tilt of the solid surface, as mentioned in the main paper. Hence, we get

$$\begin{cases} \mu \frac{U}{H^2} \sim \frac{P}{b} + \rho g \sin\alpha \\ \frac{P}{H} \sim \rho g \cos\alpha \end{cases} , \tag{S21}$$

which yields the relation

$$\frac{\mu U}{H^2} \sim \frac{\rho g}{b}(H\cos\alpha + b\sin\alpha) \,. \tag{S22}$$

Eq. $S22$ therefore shows how the viscous shear stresses balance with the hydrostatic pressure. Plugging back into Eq. S19, we get that

$$\frac{f_{\parallel,S}}{f_{\parallel,TL}} \sim \frac{b}{H}\frac{\mu U}{\gamma_{LV}} \sim \frac{\mu U}{H^2}\frac{bH}{\gamma_{LV}} \sim \frac{\rho g H}{\gamma_{LV}}(H\cos\alpha + b\sin\alpha) \sim \mathrm{Bo}\,, \tag{S23}$$

which shows that, as one might expect, the dominance of triple line forces over viscous forces is dependent on the size of the droplet, i.e. the Bond number, in this case with a characteristic length of

$$L \equiv H\sqrt{\cos\alpha + \frac{b}{H}\sin\alpha}\ . \tag{S24}$$

References

(1) Koplik, J. Frictional Force on Sliding Drops. *Phys. Rev. Fluids* **2019**, *4* (1), 014001. https://doi.org/10.1103/PhysRevFluids.4.014001.

(2) Grand, N. L.; Daerr, A.; Limat, L. Shape and Motion of Drops Sliding down an Inclined Plane. *J. Fluid Mech.* **2005**, *541*, 293–315. https://doi.org/10.1017/S0022112005006105.

(3) Limat, L. Drops Sliding down an Incline at Large Contact Line Velocity: What Happens on the Road towards Rolling? *J. Fluid Mech.* **2014**, *738*, 1–4. https://doi.org/10.1017/jfm.2013.419.

(4) Semprebon, C.; Brinkmann, M. On the Onset of Motion of Sliding Drops. *Soft Matter* **2014**, *10* (18), 3325–3334. https://doi.org/10.1039/C3SM51959G.

(5) Vretblad, A. *Fourier Analysis and Its Applications*; Axler, S., Gehring, F. W., Ribet, K. A., Eds.; Graduate Texts in Mathematics; Springer: New York, NY, 2003; Vol. 223. https://doi.org/10.1007/b97452.

(6) Wang, F.; Zhao, M. Behavior of Moving Droplet on Inclined Containment Wall: Experiment and Model Validation. *Nucl. Eng. Des.* **2021**, *376*, 111129. https://doi.org/10.1016/j.nucengdes.2021.111129.

(7) Tadmor, R. Open Problems in Wetting Phenomena: Pinning Retention Forces. *Langmuir* **2021**, *37* (21), 6357–6372. https://doi.org/10.1021/acs.langmuir.0c02768.

(8) de Gennes, P.-G.; Brochard-Wyart, F.; Quéré, D. Hysteresis and Elasticity of Triple Lines. In *Capillarity and Wetting Phenomena: Drops, Bubbles, Pearls, Waves*; de Gennes, P.-G., Brochard-Wyart, F., Quéré, D., Eds.; Springer: New York, NY, 2004; pp 69–85. https://doi.org/10.1007/978-0-387-21656-0_3.

(9) Joanny, J. F.; de Gennes, P. G. A Model for Contact Angle Hysteresis. *J. Chem. Phys.* **1984**, *81* (1), 552–562. https://doi.org/10.1063/1.447337.

(10) Pompe, T.; Herminghaus, S. Three-Phase Contact Line Energetics from Nanoscale Liquid Surface Topographies. *Phys. Rev. Lett.* **2000**, *85* (9), 1930–1933. https://doi.org/10.1103/PhysRevLett.85.1930.

(11) Decker, E. L.; Garoff, S. Contact Line Structure and Dynamics on Surfaces with Contact Angle Hysteresis. *Langmuir* **1997**, *13* (23), 6321–6332. https://doi.org/10.1021/la970528q.

(12) Landau, L. D.; Lifshitz, E. M. CHAPTER II - VISCOUS FLUIDS. In *Fluid Mechanics (Second Edition)*; Landau, L. D., Lifshitz, E. M., Eds.; Pergamon, 1987; pp 44–94. https://doi.org/10.1016/B978-0-08-033933-7.50010-6.
